# Increase in Ultrasonic Intensity of Blood Speckle across Moderate Coronary Artery Stenosis Is an Independent Predictor of Functional Coronary Artery Stenosis Measured by Fractional Flow Reserve: Pilot Study

**DOI:** 10.1371/journal.pone.0116727

**Published:** 2015-01-21

**Authors:** Jun Tanno, Shintaro Nakano, Takatoshi Kasai, Junya Ako, Sunao Nakamura, Takaaki Senbonmatsu, Shigeyuki Nishimura

**Affiliations:** 1 Department of Cardiology, Saitama Medical University, International Medical Center, Saitama, Japan; 2 New Tokyo Hospital, Chiba, Japan; 3 Department of Cardiology, Juntendo University School of Medicine, Tokyo, Japan; 4 Department of Cardiovascular Medicine, Kitasato University School of Medicine, Kanagawa, Japan; Sapienza University of Rome, ITALY

## Abstract

**Background and Aims:**

The degree of coronary artery stenosis should be assessed both anatomically and functionally. We observed that the intensity of blood speckle (IBS) on intravascular ultrasound (IVUS) is low proximal to a coronary artery stenosis, and high distal to the stenosis. We defined step-up IBS as the distal minus the proximal IBS, and speculated that this new parameter could be used for the functional evaluation of stenosis on IVUS. The aims of this study were to assess the relationships between step-up IBS and factors that affect coronary blood flow, and between step-up IBS and fractional flow reserve (FFR).

**Methods and Results:**

This study enrolled 36 consecutive patients with angina who had a single moderate stenosis in the left anterior descending artery. All patients were evaluated by integrated backscatter IVUS and intracoronary pressure measurements. FFR was calculated from measurements using a coronary pressure wire during hyperemia. Conventional gray-scale IVUS images were recorded, and integrated backscatter was measured in three cross-sectional slices proximal and distal to the stenosis. Step-up IBS was calculated as (mean distal integrated backscatter value) − (mean proximal integrated backscatter value). Stepwise multiple linear regression analysis showed that the heart rate (*r* = 0.45, *P* = 0.005), ejection fraction (*r* = −0.39, *P* = 0.01), and hemoglobin level (*r* = −0.32, *P* = 0.04) were independently correlated with step-up IBS, whereas proximal and distal IBS were not associated with these factors. There was a strong inverse correlation between step-up IBS and FFR (*r* = −0.84, *P* < 0.001), which remained significant on stepwise multiple linear regression analysis.

**Conclusions:**

The newly defined parameter of step-up IBS is potentially useful for the functional assessment of coronary artery stenosis.

## Introduction

The significance of coronary artery stenosis can be assessed both anatomically and functionally [1,2]. Anatomical stenosis can be assessed by coronary artery angiography [3], computed tomography [4], and intravascular ultrasound (IVUS) [2], and functional stenosis can be assessed by perfusion scintigraphy [5] and fractional flow reserve (FFR) [6]. Current guidelines recommend routine functional assessment of angiographically determined intermediate coronary lesions by measurement of the FFR before percutaneous coronary intervention (PCI) procedures [7,8]. In a stenotic coronary artery, FFR is defined as the ratio of the maximum achievable flow rate to the theoretical maximum flow rate. FFR-guided PCI results in better long-term outcomes than angiography-guided PCI [9].

IVUS can be used for further anatomical assessment of angiographically determined intermediate coronary stenoses [10]. We observed that the intensity of blood speckle (IBS) on IVUS is low proximal to a coronary artery stenosis, and high distal to the stenosis. This led us to consider that step-up IBS, defined as the distal minus the proximal IBS, may be related to the change in blood flow across the stenosis, which is affected by the degree of stenosis. We speculated that this new parameter may be useful for the functional evaluation of stenosis. Calculation of the step-up IBS value may therefore enable both anatomical and functional evaluation of stenosis during a single IVUS scan. However, the clinical significance of step-up IBS values is currently unclear.

We hypothesized that the step-up IBS value across a moderate coronary artery stenosis could be used to detect a functionally significant stenosis. The first part of this study assessed the relationships between step-up IBS and factors that affect coronary blood flow, and the second part assessed the relationship between step-up IBS and FFR.

## Methods

### Ethics statement

All clinical investigation was conducted according to the principles expressed in the Declaration of Helsinki. The study protocol was approved by the Institutional Review Board of Saitama Medical University International Medical Center, and written informed consent for examination including CAG, IVUS, and FFR was obtained from all patients.

### Study Population

This study included 36 consecutive patients who were diagnosed with discrete moderate stenotic lesions (30–70% diameter stenosis on visual estimation) of the left anterior descending (LAD) artery on coronary angiography between June 2009 and June 2011. Six patients were excluded because of tandem lesions or missing IVUS data, and data from the remaining 30 patients were analyzed. All included patients underwent integrated backscatter IVUS (IB-IVUS) and intracoronary pressure measurements at the time of diagnostic catheterization. This study was the post-hoc analysis of the pooled IVUS data, which was primarily made to investigate the objectives of this study.

### Angiographic Analysis

Quantitative coronary angiography analyses were performed by an experienced independent observer blinded to the FFR, IB-IVUS, and clinical data, using the Cardiovascular Angiography Analysis System II (Pie Medical Imaging BV, Maastricht, The Netherlands). The computer-defined minimum lumen diameter, reference vessel diameter (via the interpolation method), and percent diameter stenosis were recorded [3].

### FFR Measurement

FFR was measured using a coronary pressure guidewire (Radi; St. Jude Medical, Inc., Uppsala, Sweden) during maximal hyperemia, induced by intravenous administration of adenosine triphosphate disodium hydrate at 150 μg/kg/min via a peripheral vein [14,15]. Equalization was achieved with the guidewire sensor positioned at the guiding catheter tip. FFR was calculated as the mean distal coronary artery pressure (measured by the pressure guidewire) divided by the mean aortic pressure (simultaneously measured by the guiding catheter) during maximal hyperemia. An FFR value of ≤0.80 was considered pathologically significant [7].

### Conventional IVUS and IB-IVUS Analyses

All IVUS analyses were performed by an experienced analyst who was blinded to the angiographic findings, the FFR, clinical data, and lesion characteristics. The cross-sectional areas (CSAs) of the external elastic membrane and lumen were measured, and the CSA of the plaque was calculated as (CSA of external elastic membrane) − (CSA of lumen). The average CSAs of the external elastic membrane, lumen, and plaque of each target lesion were calculated according to the criteria of the clinical expert consensus document on IVUS [16]. Manual tracing was performed for each 0.5-mm cross-section, and two manually traced images were automatically interpolated using the IVUS imaging system (VISIWAVE; Terumo Corp., Tokyo, Japan).

**Figure 1:**
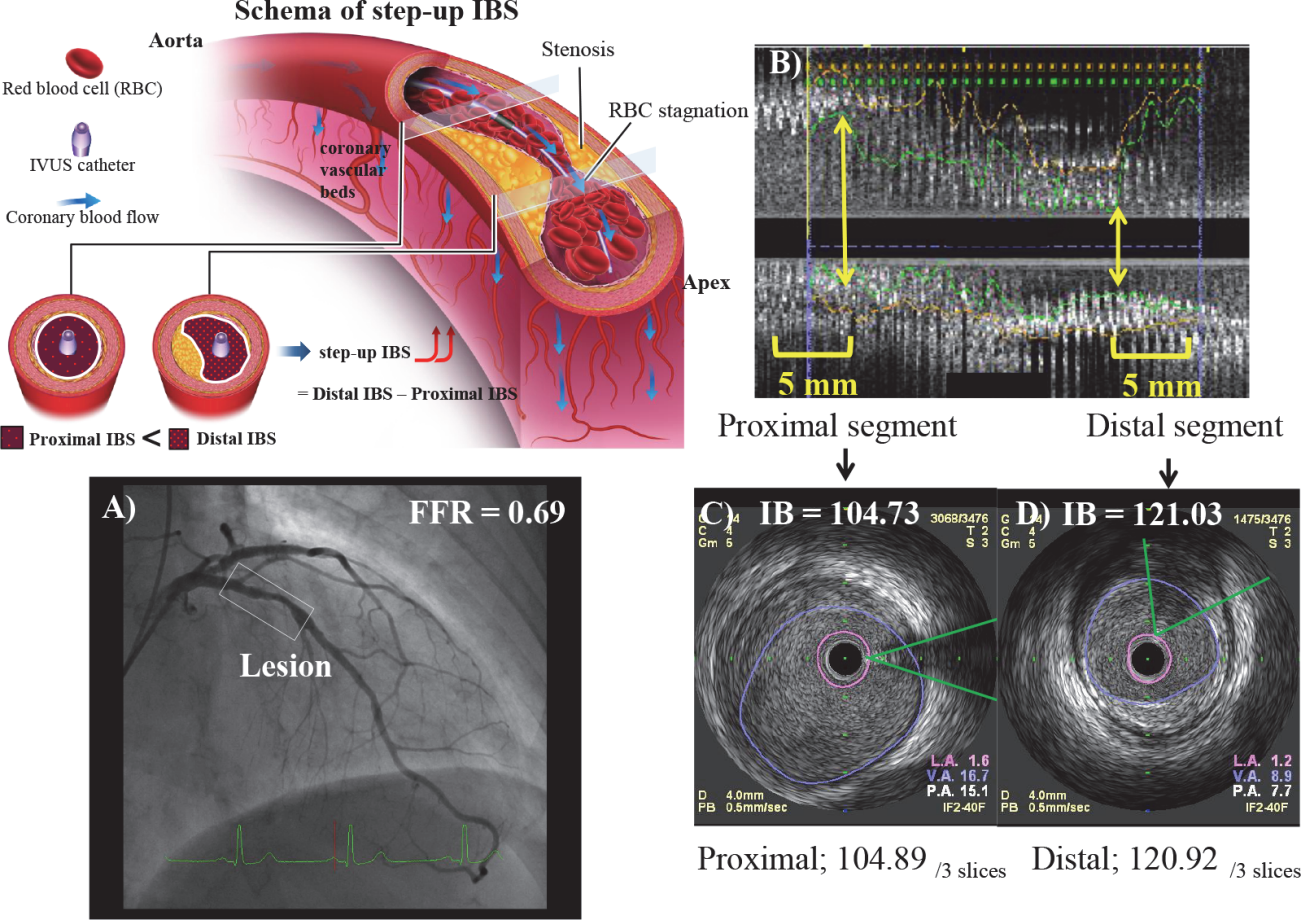
Schema of Step-up IBS and Calculation of Step-up IBS. (Schema of Step-up IBS) Schema of the concepts and methods for measuring step-up IBS. (A) Coronary angiography, showing a discrete moderate stenotic lesion of the LAD with an FFR value of 0.69. (B) Longitudinal IVUS image of the lesion. The proximal IBS value was measured in a segment (proximal segment) 5 mm proximal to the site with the largest lumen proximal to a stenosis but within the target lesion. The distal IBS value was measured in a segment (distal segment) 5 mm distal to the site of the smallest lumen size within the target lesion (C and D) Manually encircled cross-sectional vessel lumens and integrated backscatter values measured in the proximal segment (C) and distal segment (D) using the IB-IVUS imaging system in the end-diastolic frame. The acoustic shadows of the guidewire were manually traced and excluded to minimize acoustic artifacts. The proximal and distal integrated backscatter values were 104.73 and 121.03, respectively. Integrated backscatter values were measured in three cross-sectional slices proximal and distal to the target lesion. The mean integrated backscatter values at the proximal and distal sites were 104.89 and 120.92, respectively, and the step-up IBS value was 16.03. LAD, left anterior descending artery; FFR, fractional flow reserve; IVUS, intravascular ultrasound; IBS, intensity of blood speckle; IB-IVUS, integrated backscatter intravascular ultrasound.

IB-IVUS data were recorded on the imaging system hard drive. A personal computer equipped with custom software (VISIATRAS; Terumo Corp.) was connected to the IVUS imaging system to obtain a radiofrequency signal output, signal trigger output, and video image output. The proximal IBS value was measured in a segment (proximal segment) 5 mm proximal to the site with the largest lumen proximal to a stenosis but within the target lesion. The distal IBS value was measured in a segment (distal segment) 5 mm distal to the site of the smallest lumen size within the target lesion. (Fig. 1) [11]. IBS values were obtained in the end-diastolic frame during basal conditions (without hyperemia). Integrated backscatter values were measured in three cross-sectional slices per segment, and the mean value for each segment was used as the IBS value for the analyses. Step-up IBS was defined as (mean distal integrated backscatter value) − (mean proximal integrated backscatter value) (Fig. 1).

### Statistical Analyses

All statistical analyses were performed using SPSS statistical software (version 18.0; SPSS Inc., Chicago, IL, USA). Continuous variables are expressed as mean ± standard deviation, and categorical variables are expressed as frequency and percentage. The relationships between variables were analyzed using Pearson’s correlation coefficient, and the relationships between FFR or step-up IBS and independent variables were analyzed using stepwise multiple linear regression analysis. Variables with a probability (*P*) value of <0.05 were included and variables with a *P* value of >0.10 were excluded. The multiple linear regression analysis for factors related to step-up IBS included age, sex, body mass index, ejection fraction (EF), hemoglobin level, creatinine level, mean blood pressure during the investigation, heart rate during the investigation, percent diameter stenosis, lesion length, minimum lesion area, maximum lesion area, minimum vessel area, maximum vessel area, lumen volume, vessel volume, plaque volume, percent plaque volume, and proximal and distal IBS. The multiple linear regression analysis for factors related to FFR included the same variables as well as step-up IBS. Collinearity was verified for each pair of variables. A *P* value of <0.05 was considered statistically significant for all analyses.

Reproducibility was assessed by the intraobserver correlation coefficient and the coefficient of variation for two calculations of the step-up IBS value.

## Results

### Patient Characteristics and Percent Diameter Stenosis

Thirty discrete LAD artery lesions were evaluated in 30 patients (25 males and 5 females). The patient characteristics and percent diameter stenosis as determined by quantitative coronary angiography are shown in Table 1. Most patients were elderly, nonobese men with preserved left ventricular systolic function. Six (20%) patients had a history of myocardial infarction, but none had a history of myocardial infarction during the 6 weeks prior to the assessment. The mean percent diameter stenosis was 46.8 ± 8.3% (range, 31.8–68.0%).

**Table 1:** Patient characteristics and percent diameter stenosis by QCA analysis.

Mean age, years	65.7 ± 9.1
Male, %	83.3 (25/30)
Weight, kg	64.1 ± 12.2
Height, cm	163.52 ± 8.8
BM, kg/m^2^	24.1 ± 3.1
Diabetes mellitus, %	46.7 (14/30)
Hypertension, %	76.7 (23/30)
Dyslipidemia, %	93.3 (28/30)
Current smoker, %	36.7 (11/30)
Family history, %	13.3 (4/30)
Previous MI, %	20.0 (6/30)
Systolic blood pressure, mmHg	147.4 ± 21.5
Diastolic blood pressure, mmHg	76.8 ± 12.6
Heart rate, beats/min	70.0 ± 13.8
EF, %	60.8 ± 6.9
Hb, g/dl	13.4 ± 2.1
Cr, mg/dl	1.3 ± 1.8
Percent diameter stenosis (QCA), %	46.8 ± 10.2

Continuous variables are expressed as mean ± SD, and categorical variables are expressed as number (%). BMI, body mass index; EF, ejection fraction; Hb, hemoglobin; Cr, creatinine; QCA, quantitative coronary angiography.

### Measurement of FFR and IVUS Parameters

Coronary pressure was successfully measured in all 30 patients without serious complications. The mean FFR value was 0.77 ± 0.09 (range, 0.62–0.93). Twenty lesions were associated with an FFR value of ≤0.80. The IVUS parameters are shown in Table 2. The mean lesion length was 31.2 ± 12.3 mm, and the mean minimum lumen area was 2.8 ± 0.9 mm^2^. The proximal, distal, and step-up IBS values are also shown in Table 2. For step-up IBS values, the intraobserver correlation coefficient was 0.99 and the coefficient of variation was 4.9.

**Table 2:** IVUS parameters.

Lesion length, mm	31.2 ± 12.3
Minimum lumen area, mm^2^	2.8 ± 0.94
Maximum lumen area, mm^2^	10.0 ± 3.1
Minimum vessel area, mm^2^	8.5 ± 2.9
Maximum vessel area, mm^2^	17.5 ± 4.6
Lumen volume, mm^3^	177.6 ± 79.4
Vessel volume, mm^3^	386.8 ± 164.1
Plaque volume, mm^3^	209.1 ± 97.4
% plaque volume	53.3 ± 8.0
Proximal, integrated backscatter value	100.2 ± 14.4
Distal, integrated backscatter value	113.9 ± 14.4
Step-up IBS, IB value	13.8 ± 8.1

Continuous variables are expressed as mean ± SD.

IBS, intensity of blood speckle; IVUS, intravascular ultrasound.

### Factors Correlating with the Proximal, Distal, and Step-Up IBS Values

Univariate analyses did not show any significant correlations between proximal and distal IBS and patient characteristics or individual IVUS parameters (Table 3). However, there were significant correlations between step-up IBS and age, EF, heart rate, and IVUS parameters (maximum lumen area and maximum vessel area) (Table 3). Stepwise multiple linear regression analysis showed that increased heart rate, decreased EF, and decreased hemoglobin level were significantly and independently correlated with step-up IBS (Table 4).

**Table 3:** Correlations with proximal, distal, and step-up IBS values.

	**Proximal IBS**		**Distal IBS**		**Step-up IBS**	
	**Coefficient (r)**	**P**	**Coefficient (r)**	**P**	**Coefficient (r)**	**P**
Age	0.17	0.36	−0.06	0.77	−0.41	0.03
Male	0.18	0.33	0.07	0.71	−0.20	0.30
BMI	0.11	0.57	0.10	0.61	−0.02	0.91
EF	0.28	0.13	0.07	0.71	−0.37	0.04
Hb	−0.09	0.64	−0.25	0.18	−0.29	0.12
Cr	0.13	0.50	0.17	0.38	0.07	0.71
Mean blood pressure	−0.12	0.55	0.05	0.81	0.29	0.13
Heart rates	0.08	0.68	0.33	0.08	0.44	0.01
Percent diameter stenosis	0.02	0.92	0.16	0.40	0.25	0.18
Lesion length	0.09	0.62	0.26	0.17	0.30	0.11
Minimum lumen area	−0.17	0.37	−0.28	0.14	−0.19	0.31
Maximum lumen area	−0.16	0.40	0.05	0.78	0.38	0.04
Minimum vessel area	−0.19	0.32	−0.23	0.22	−0.08	0.67
Maximum vessel area	−0.13	0.49	0.01	0.60	0.41	0.02
Lumen volume	0.04	0.99	0.13	0.48	0.24	0.21
Vessel volume	0.09	0.65	0.23	0.23	0.25	0.18
Plaque volume	0.14	0.45	0.27	0.15	0.23	0.23
Percent plaque volume	0.20	0.29	0.20	0.29	0.002	0.99
Proximal IBS	-	-	0.84	<0.001	−0.27	0.14
Distal IBS	0.84	<0.001	-	-	0.29	0.13
Step-up IBS	−0.27	0.14	0.29	0.13	-	-

BMI, body mass index; EF, ejection fraction; Hb, hemoglobin; Cr, creatinine; QCA, quantitative coronary angiography; IBS, intensity of blood speckle.

**Table 4:** Multiple stepwise linear regression analysis of relationships between step-up IBS and independent variables.

**Independent variables**	**Correlation coefficient (r)**	**R^2^**	***P***
Heart rates	0.45	0.20	0.005
EF	−0.39	0.15	0.01
Hb	−0.32	0.10	0.04
Total	0.66	0.44	0.01

EF, ejection fraction; Hb, hemoglobin.

### Correlations between FFR and Step-Up IBS

**Figure 2:**
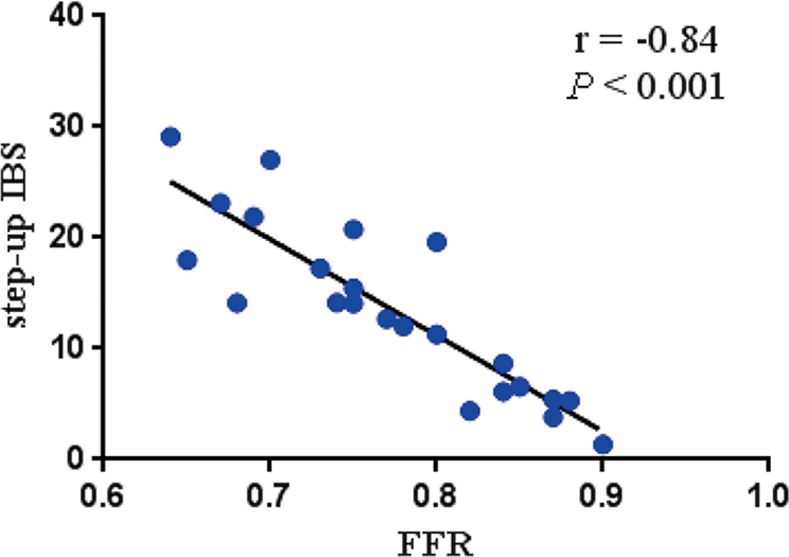
Correlation between FFR and step-up IBS. There was an inverse correlation between FFR and step-up IBS (*r* = −0.84; *R^2^* = 0.71; *P* < 0.001). FFR, fractional flow reserve; IBS, intensity of blood speckle.

As shown in Table 5, there were no significant correlations between FFR and patient characteristics, except for an inverse correlation with percent diameter stenosis (*r* = −0.37, *R^2^* = 0.14, *P* = 0.040). There were also no significant correlations between FFR and the proximal and distal IBS. Univariate analyses did not show significant correlations between FFR and individual IVUS parameters. However, there was a significant inverse correlation between FFR and step-up IBS (*r* = −0.84, *R^2^* = 0.71, *P* < 0.001) (Fig. 2). Stepwise multiple linear regression analysis including FFR as a dependent variable showed that only step-up IBS (*R^2^* = 0.71, *P* < 0.001) was significantly and independently correlated with FFR.

**Table 5:** Univariate analyses of relationships between FFR and independent variables.

	**Correlation coefficient(r)**	**R^2^**	***P***
Age	0.34	0.12	0.07
Male	0.32	0.10	0.08
BMI	0.03	0.001	0.89
EF	0.34	0.12	0.06
Hb	0.33	0.11	0.07
Cr	−0.20	0.04	0.30
Mean blood pressure	−0.19	0.04	0.32
Heart rates	−0.32	0.10	0.08
Percent diameter stenosis	−0.37	0.14	0.04
Lesion length	−0.24	0.06	0.21
Minimum lumen area	0.29	0.08	0.12
Maximum lumen area	−0.15	0.02	0.44
Minimum vessel area	0.14	0.02	0.47
Maximum vessel area	−0.17	0.03	0.36
Lumen volume	−0.11	0.01	0.55
Vessel volume	−0.17	0.03	0.37
Plaque volume	−0.19	0.04	0.31
Percent plaque volume	−0.18	0.03	0.35
Proximal IBS	0.17	0.03	0.38
Distal IBS	−0.31	0.10	0.11
Step-up IBS	−0.84	0.71	<0.001

BMI, body mass index; EF, ejection fraction; Hb, hemoglobin; Cr, creatinine; QCA, quantitative coronary angiography; IBS, intensity of blood speckle.

## Discussion

The major findings of the present study are as follows: (1) stepwise multiple linear regression analysis showed that step-up IBS was associated with factors that can affect coronary blood flow, such as heart rate, EF, and hemoglobin level, whereas proximal and distal IBS were not associated with these factors; and (2) there was a significant relationship between step-up IBS and FFR, and the only factor significantly correlated with FFR on stepwise multiple linear regression analysis was step-up IBS. These findings suggest that step-up IBS (but not proximal or distal IBS) may be an indicator of coronary blood flow, and that step-up IBS may be useful for the functional assessment of coronary artery stenosis, similar to FFR.

### Factors Correlated with Proximal, Distal, and Step-Up IBS

Proximal and distal IBS values are not considered to reflect coronary blood flow because coronary blood flow is not an absolute value, but is determined by the difference between the entrance pressure and the pressure in the coronary vascular bed [17]. We hypothesized that the IVUS catheter affects both the proximal and distal IBS, and that this influence may be minimized by calculating the difference between the proximal and distal IBS. In this study, there were no significant correlations between factors that influence coronary blood flow and the proximal and distal IBS values.

The step-up IBS value can be explained by alterations in blood properties that are mainly associated with red blood cell (RBC) stagnation [11]. van der Heiden et al. [12] clearly demonstrated that ultrasound backscatter in human blood is caused by RBCs, and that integrated backscatter power increases with decreasing shear rates and increasing aggregation (rouleaux) size. These findings suggest that the greater the blood viscosity, the greater the integrated backscatter power, considering that blood viscosity is inversely related to shear rate [18].

In this study, step-up IBS was associated with factors that affect coronary blood flow, such as heart rate, EF, and hemoglobin level, independent of the degree of coronary artery stenosis. When the heart rate increases, the coronary blood flow decreases because of the higher proportion of time spent in systole [19]. Increased heart rate may therefore be directly associated with increased step-up IBS values. Increased left ventricular diastolic filling pressure decreases coronary blood flow because it compresses the intramural coronary vasculature, thereby reducing the perfusion pressure of the coronary bed [20]. Patients with decreased left ventricular EF are likely to have increased left ventricular filling pressure, resulting in reduced coronary blood flow and increased step-up IBS. In patients with anemia, compensatory reactions such as increased heart rate and increased contraction protect against the decreased oxygen-carrying capacity of the blood to the myocardium. These reactions increase extravascular compressive forces, resulting in functional limitation of coronary blood flow [17].

### Correlation between Step-Up IBS and FFR

As described above, step-up IBS is determined by the decrease in coronary blood flow across a stenotic lesion during basal conditions (i.e., without hyperemia). Basically, myocardial ischemia is caused by an imbalance between myocardial oxygen consumption and supply [21,22]. When there is insufficient oxygen supply to the myocardium during basal conditions due to moderate epicardial coronary stenosis, the autoregulation system preserves the blood flow to the myocardium relative to the entrance flow [17]. However, flow decreases between the inlet region (proximal to the stenosis) and the outlet region (distal to the stenosis) [13]. The coronary blood velocity (i.e., shear rate) will therefore be lower in the outlet region than in the inlet region during basal conditions, and there will be a relative increase in blood viscosity at the outlet of the stenosis because of enhanced interactions among RBCs (RBC stagnation), and because blood behaves as a non-Newtonian fluid when coronary flow decreases [13]. The increase in integrated backscatter power (step-up IBS) across a stenotic lesion is therefore related to the characteristics of blood flow, especially RBC stagnation because of decreased blood flow (Fig. 1).

FFR is the ratio of the coronary pressure distal to a moderate epicardial coronary stenosis to the entrance pressure (i.e., aortic pressure) during hyperemia. There is a liner relationship between coronary perfusion pressure and myocardial blood flow (i.e., oxygen supply) during hyperemia [23]. The greater the reduction in perfusion pressure (i.e., the smaller the FFR), the slower the myocardial blood flow. Both step-up IBS and FFR can therefore reflect the poststenotic decrease in blood flow in a coronary artery with moderate stenosis, thereby indicating the degree of insufficiency of oxygen supply to the myocardium, even though they are determined under different conditions (basal and hyperemic, respectively). In this study, step-up IBS was significantly correlated with FFR, and this relationship remained significant on stepwise multiple linear regression analysis.

### Clinical Implications

Coronary artery evaluation using IVUS helps to determine lesion morphology, and to select appropriate devices, balloon sizes, and stent geometry. The results of the present study suggest that measurement of step-up IBS in conjunction with standard anatomical assessment of the coronary artery by IVUS has the potential to assess both the morphological and physiological severity of coronary stenotic lesions using a single device, without the need to introduce a coronary pressure guidewire, and may substantially reduce the overall treatment cost and shorten the intervention time.

### Limitations

This study has some limitations. First, the number of patients was limited because only those presenting with moderate (30–70%) LAD artery stenosis were included. However, the number of included patients was sufficient to show the clear relationship between step-up IBS and FFR. Second, only patients with moderate stenosis were included because recent guidelines recommend the use of FFR to assess intermediate coronary lesions determined by angiography [10,24]. Third, to decrease selection bias, only patients with IVUS data and LAD artery lesions were included. Patients with stenotic lesions caused by bending or small vessel size were therefore automatically excluded. Fourth, none of the patients in this study had a major bifurcation of the coronary artery near the IBS measurement site, and the effects of such a bifurcation are therefore unclear. This was a proof-of-concept study aimed at assessing the potential clinical utility of step-up IBS values, and larger clinical studies are required to assess step-up IBS values in other coronary arteries and in the presence of bifurcation of the coronary artery. Fifth, all patients in the study were taking dual antiplatelet agents (aspirin plus clopidogrel) at the time of measurement of step-up IBS, and the effects of such platelet inhibitors are therefore unclear in this study. However, in clinical settings, patients with angina who will undergo IVUS usually have been taking antiplatelet agents. Finally, step-up IBS was measured during basal conditions and not hyperemic conditions. Further studies comparing instantaneous wave-free ratio and step-up IBS are required. Moreover, prospective studies are required to validate the selected step-up IBS cut-off value and assess the relationship between the cut-off value and long-term clinical outcomes.

### Conclusions

This study identified a significant relationship between step-up IBS and FFR. Multiple linear regression analysis showed that step-up IBS had a significant, independent, inverse correlation with FFR. Our findings suggest that step-up IBS, a novel parameter based on IVUS findings without induced hyperemia, may be useful for assessment of the physiological severity of lesions

## References

[1] Pakkal M, Raj V, McCann GP (2011) Non-invasive imaging in coronary artery disease including anatomical and functional evaluation of ischaemia and viability assessment. Br J Radiol 84 Spec No 3: S280–295. 10.1259/bjr/50903757 22723535 PMC3473910

[2] Tobis J, Azarbal B, Slavin L (2007) Assessment of intermediate severity coronary lesions in the catheterization laboratory. J Am Coll Cardiol 49: 839–848. 10.1016/j.jacc.2006.10.055 17320741

[3] Scanlon PJ, Faxon DP, Audet AM, Carabello B, Dehmer GJ, et al. (1999) ACC/AHA guidelines for coronary angiography. A report of the American College of Cardiology/American Heart Association Task Force on practice guidelines (Committee on Coronary Angiography). Developed in collaboration with the Society for Cardiac Angiography and Interventions. J Am Coll Cardiol 33: 1756–1824. 10.1016/S0735-1097(99)00126-6 10334456

[4] Cho I, Chang HJ, Sung JM, Pencina MJ, Lin FY, et al. (2012) Coronary computed tomographic angiography and risk of all-cause mortality and nonfatal myocardial infarction in subjects without chest pain syndrome from the CONFIRM Registry (coronary CT angiography evaluation for clinical outcomes: an international multicenter registry). Circulation 126: 304–313. 10.1161/CIRCULATIONAHA.111.081380 22685117

[5] Gibbons RJ, Balady GJ, Bricker JT, Chaitman BR, Fletcher GF, et al. (2002) ACC/AHA 2002 guideline update for exercise testing: summary article. A report of the American College of Cardiology/American Heart Association Task Force on Practice Guidelines (Committee to Update the 1997 Exercise Testing Guidelines). J Am Coll Cardiol 40: 1531–1540. 10.1016/S0735-1097(02)02164-2 12392846

[6] Pijls NH, De Bruyne B, Peels K, Van Der Voort PH, Bonnier HJ, et al. (1996) Measurement of fractional flow reserve to assess the functional severity of coronary-artery stenoses. N Engl J Med 334: 1703–1708. 10.1056/NEJM199606273342604 8637515

[7] Levine GN, Bates ER, Blankenship JC, Bailey SR, Bittl JA, et al. (2011) 2011 ACCF/AHA/SCAI Guideline for Percutaneous Coronary Intervention: a report of the American College of Cardiology Foundation/American Heart Association Task Force on Practice Guidelines and the Society for Cardiovascular Angiography and Interventions. Circulation 124: e574–651. 10.1161/CIR.0b013e31823ba622 22064601

[8] Wijns W, Kolh P, Danchin N, Di Mario C, Falk V, et al. (2010) Guidelines on myocardial revascularization. Eur Heart J 31: 2501–2555. 10.1093/eurheartj/ehq277 20802248

[9] Tonino PA, De Bruyne B, Pijls NH, Siebert U, Ikeno F, et al. (2009) Fractional flow reserve versus angiography for guiding percutaneous coronary intervention. N Engl J Med 360: 213–224. 10.1056/NEJMoa0807611 19144937

[10] Levine GN, Bates ER, Blankenship JC, Bailey SR, Bittl JA, et al. (2011) 2011 ACCF/AHA/SCAI Guideline for Percutaneous Coronary Intervention: executive summary: a report of the American College of Cardiology Foundation/American Heart Association Task Force on Practice Guidelines and the Society for Cardiovascular Angiography and Interventions. Circulation 124: 2574–2609. 10.1161/CIR.0b013e31823a5596 22064598

[11] Mintz GS, Nissen SE, Anderson WD, Bailey SR, Erbel R, et al. (2001) American College of Cardiology Clinical Expert Consensus Document on Standards for Acquisition, Measurement and Reporting of Intravascular Ultrasound Studies (IVUS). A report of the American College of Cardiology Task Force on Clinical Expert Consensus Documents. J Am Coll Cardiol 37: 1478–1492. 10.1016/S0735-1097(01)01175-5 11300468

[12] van der Heiden MS, de Kroon MG, Bom N, Borst C (1995) Ultrasound backscatter at 30 MHz from human blood: influence of rouleau size affected by blood modification and shear rate. Ultrasound Med Biol 21: 817–826. 10.1016/0301-5629(95)00012-G 8571469

[13] Govindaraju K, Badruddin IA, Viswanathan GN, Ramesh SV, Badarudin A (2012) Evaluation of functional severity of coronary artery disease and fluid dynamics′ influence on hemodynamic parameters: A review. Phys Med. 10.1016/j.ejmp.2012.03.008 22704601

[14] Jeremias A, Filardo SD, Whitbourn RJ, Kernoff RS, Yeung AC, et al. (2000) Effects of intravenous and intracoronary adenosine 5′-triphosphate as compared with adenosine on coronary flow and pressure dynamics. Circulation 101: 318–323. 10.1161/01.CIR.101.3.318 10645929

[15] Matsuo H, Watanabe S, Kadosaki T, Yamaki T, Tanaka S, et al. (2002) Validation of collateral fractional flow reserve by myocardial perfusion imaging. Circulation 105: 1060–1065. 10.1161/hc0902.104719 11877355

[16] Mintz GS, Garcia-Garcia HM, Nicholls SJ, Weissman NJ, Bruining N, et al. (2011) Clinical expert consensus document on standards for acquisition, measurement and reporting of intravascular ultrasound regression/progression studies. EuroIntervention 6: 1123–1130, 1129. 10.4244/EIJV6I9A195 21518687

[17] Duncker DJ, Bache RJ (2008) Regulation of coronary blood flow during exercise. Physiol Rev 88: 1009–1086. 10.1152/physrev.00045.2006 18626066

[18] Ercan M, Koksal C (2003) The relationship between shear rate and vessel diameter. Anesth Analg 96: 307; author reply 307-308. 10.1097/00000539-200301000-00073 12505978

[19] Bache RJ, Cobb FR (1977) Effect of maximal coronary vasodilation on transmural myocardial perfusion during tachycardia in the awake dog. Circ Res 41: 648–653. 10.1161/01.RES.41.5.648 332406

[20] Ellis AK, Klocke FJ (1980) Effects of preload on the transmural distribution of perfusion and pressure-flow relationships in the canine coronary vascular bed. Circ Res 46: 68–77. 10.1161/01.RES.46.1.68 7349919

[21] Braunwald E (1971) Control of myocardial oxygen consumption: physiologic and clinical considerations. Am J Cardiol 27: 416–432. 10.1016/0002-9149(71)90439-5 4396726

[22] Crossman DC (2004) The pathophysiology of myocardial ischaemia. Heart 90: 576–580. 10.1136/hrt.2003.029017 15084567 PMC1768241

[23] Pijls NH, Sels JW (2012) Functional measurement of coronary stenosis. J Am Coll Cardiol 59: 1045–1057. 10.1016/j.jacc.2011.09.077 22421298

[24] Hillis LD, Smith PK, Anderson JL, Bittl JA, Bridges CR, et al. (2011) 2011 ACCF/AHA Guideline for Coronary Artery Bypass Graft Surgery: a report of the American College of Cardiology Foundation/American Heart Association Task Force on Practice Guidelines. Circulation 124: e652–735. 10.1161/CIR.0b013e31823c074e 22064599

